# Oral function is associated with the body and muscle mass indices of middle‐aged dental patients

**DOI:** 10.1002/cre2.514

**Published:** 2021-11-17

**Authors:** Tomotaka Nishi, Midori Ohta, Tomofumi Takano, Koichiro Ogami, Takayuki Ueda, Kaoru Sakurai

**Affiliations:** ^1^ Department of Removable Prosthodontics & Gerodontology Tokyo Dental College Tokyo Japan; ^2^ Chiba Dental Center Tokyo Dental College Chiba Japan

**Keywords:** body mass index, middle aged, oral health, undernutrition

## Abstract

**Objectives:**

Undernutrition is associated with frailty, one of the common reasons for seeking long‐term care. In older adults, nutritional status is known to be associated with oral function; however, it is not yet known if there is a similar association in middle‐aged adults. The present study determined the association between nutritional status and oral function in middle‐aged adults.

**Material and methods:**

This cross‐sectional study was conducted in 117 outpatients aged 40–64 years who underwent dental check‐up at a private dental clinic. Factors associated with oral function, including oral moisture, number of teeth present, oral diadochokinesis, tongue pressure, lip‐seal strength, and masticatory performance, were evaluated. Body mass index (BMI), fat‐free mass index (FFMI), and skeletal muscle mass index (SMI) were analyzed as nutritional statuses. Pearson's correlation analysis was conducted to determine the correlation between nutritional statuses and other variables. Multiple linear regression analysis was performed, with adjustment for age and sex, using BMI, FFMI, or SMI as response variables and oral moisture, maximum tongue pressure, lip‐seal strength, oral diadochokinetic rate, and masticatory performance as explanatory variables.

**Results:**

All nutritional statuses showed significant correlation with maximum tongue pressure and lip‐seal strength. Multiple linear regression analysis revealed that BMI was associated with maximum tongue pressure and lip‐seal strength, whereas FFMI and SMI showed significant association with sex, maximum tongue pressure, and lip‐seal strength.

**Conclusions:**

In middle‐aged outpatients at a regional dental clinic, lip‐seal strength and tongue pressure were positively associated with BMI, FFMI, and SMI.

## 1 INTRODUCTION

There has been an increase in the aging population worldwide, with greater importance being given to the extension of healthy life expectancy, which requires the prevention of reaching a health state that requires long‐term care. When an older adult starts requiring long‐term care, it becomes difficult for them to manage without it. Therefore, it is important to prevent adults from reaching that state from an early stage of deterioration. In Japan, frailty has been reported to be the third‐most frequent cause of requiring long‐term care (Cabinet Office, 2016). As physical frailty progresses, the person reaches a state of undernutrition and requires long‐term care (Fried et al., 2001; Xue et al., 2008). In Japan, oral hypofunction is defined as a disorder in which oral functions are reduced in a complex manner (Minakuchi et al., 2018). Therefore, multiple functions have been comprehensively evaluated instead of individual evaluation of oral functions to diagnose oral hypofunction. Oral functions include motor, secretory, and sensory functions. Oral motor functions involve occlusal force, tongue function, lip function, masticatory function, and swallowing function. Oral secretory function comprises salivary secretion, whereas oral sensory function includes taste. Studies have reported that some oral motor and secretory functions, such as the number of teeth present (Kikutani et al., 2013), occlusal force (Inomata et al., 2014), masticatory function (Ikebe et al., 2006), and salivary secretion (Samnieng, 2015), are associated with the nutritional status of older adults. Reduced oral function similar to that in older adults has been observed in middle‐aged adults (Ohta et al., 2018). Furthermore, there are as many adults in their 40s and 50s as older adults with a body mass index (BMI) of ≤18.5 kg/m^2^ (Ministry of Health, Labour and Welfare, 2020). However, the association between nutritional status and oral function in middle‐aged adults has not yet been elucidated. In the case that reduced oral function is associated with undernutrition in middle‐aged adults, as observed in older adults, it is believed that evaluating oral function and its management via oral rehabilitation in middle‐aged adults with reduced oral function could prevent the decline of nutritional status at an advanced age.

The present study was conducted to explore the association between nutritional status and oral function and identify the factors related to oral function that are in turn associated with the nutritional status of middle‐aged adults attending a regional clinic. The null hypothesis was that there is no association between any oral function and the nutritional status of middle‐aged adults.

## 2 METHODS

### 2.1 Study design

This retrospective study was conducted using existing medical records from a dental clinic. We extracted the data of all subjects aged 40–64 years who underwent a dental check‐up at a regional dental clinic from July 2016 to June 2018 (Edogawa‐city, Tokyo) and subsequently performed a cross‐sectional analysis.

### 2.2 Subjects

In total, 124 patients aged 40–64 years underwent a dental check‐up at the regional dental clinic. Patients with the following conditions were excluded (exclusion criteria): acute dental and oral problems, diabetes mellitus, endocrine disease, inflammatory disease (major infections, burns, trauma, and closed head injury), gastrointestinal disease (intestinal obstruction, ischemic colitis, and gastroduodenal ulcer), and dysphagia (as determined by a repetitive saliva swallowing test (Oguchi, Saitoh, Baba, et al., 2000; Oguchi, Saitoh, Mizuno, et al., 2000) result of ≤2 times/30 s). Of the 124 patients, 117 (men: 44, women: 73, mean age: 50 ± 7 years) were included in the analysis, and 7 with missing measurement data were excluded (response rate: 94%).

The present study was performed following approval from the Ethics Committee of Tokyo Dental College (approval number: 851).

### 2.3 Measurements

Three certified examiners evaluated the factors associated with the oral function and nutritional status of the subjects. We prepared the measurement protocol, trained each examiner accordingly, and confirmed that the same measurements were performed by each examiner.

#### 2.3.1 Demographic variables

Age and sex were considered as demographic variables.

#### 2.3.2 Factors related to oral function

We adopted oral moisture as oral secretory function, whereas the movement and force of the tongue and lip, the number of remaining teeth as an alternative index of occlusal force, masticatory performance, and swallowing function regarded as oral motor functions were adopted to assess oral hypofunction. Medical equipment typically used for the measurement in Japan was used to evaluate each function. Subjects using removable dentures wore them during the measurements.

Oral moisture was measured using an oral moisture‐checking device (Mucus; Life Co., Ltd., Saitama, Japan; Fukushima et al., 2017). Mucosal wetness in the central region of the tongue dorsum at 10 mm from the tip of the tongue was considered as oral moisture. A sensor cover was placed over the oral moisture‐checking device, and the sensor was pressed uniformly to the test tongue surface with a 200 gf force for 3 s. The measurement was repeated thrice, and the median was adopted as the measured value.

The subjects repeatedly pronounced /pa/, /ta/, and /ka/ syllables for 5 s each, and the oral diadochokinetic rate per second was measured using an automatic measurement device (KENKOU‐KUN handy; Takei Scientific Instruments Co., Ltd., Niigata, Japan; Yamada et al., 2015). The measurement was repeated for three cycles. Each cycle involved repeating the syllable for 5 s each in the order of /pa/, /ta/, and /ka/. The maximum value of oral diadochokinetic rate per second was adopted as the measured value.

Maximum tongue pressure was measured using a tongue pressure measurement device (JMS tongue pressure measuring device, JMS Co., Ltd., Hiroshima, Japan; Tsuga et al., 2011). In the anterior region of the palate, the balloon of the tongue pressure probe was compressed voluntarily with the tongue using maximum force for several seconds, and the pressure was measured as the maximum tongue pressure. The measurement was repeated thrice, and the maximum value was adopted as the measured value.

Lip‐seal strength was measured using a lip‐seal strength‐measuring device (Lipplekun; SHOFU, Inc., Kyoto, Japan; Sakai et al., 2019) with the subject sitting on a chair with their head positioned such that the Frankfort plane was parallel to the floor. For the measurement, a button (Lipple button, SHOFU, Inc.) was placed in the median oral vestibule and pulled in parallel to the floor in the sagittal direction. Pulling was continued with increasing force until the button left the lip. The measurement was repeated twice, and the maximum value was adopted as the measured value.

Masticatory performance as masticatory function was measured using GLUCO SENSOR GS‐II (GC Corp., Tokyo, Japan; Uesugi & Shiga, 2017). Subjects chewed a piece of 2 g gummy jelly for 20 s and rinsed out the jelly from their mouth lightly with 10 ml of water, after which the eluate glucose concentration was measured.

#### 2.3.3 Nutritional status

Subjects' height (m), body weight (kg), fat‐free mass (FFM) (kg), and skeletal muscle mass (SM) (kg) were measured using InBody J10 (Biospace Inc., Seoul, Korea), which is capable of performing body composition analysis using Multi‐frequency bioelectrical impedance analysis (MF‐BIA) (Yamada et al., 2017) . The measurements were performed ≥1 h after bath, meals, and exercise. Other measurement conditions were in accordance with the guidelines of the European Society for Clinical Nutrition and Metabolism (ESPEN; Kyle et al., 2004). We calculated BMI (kg/m^2^) from body weight/height^2^ (Fried et al., 2001), FFMI (kg/m^2^) from FFM/height^2^ (Fried et al., 2001), and SMI (kg/m^2^) from SM/height^2^ (Fried et al., 2001). FFM is calculated by subtracting fat mass from total body weight. FFM and SM are estimated using body composition analysis.

### 2.4 Statistical analysis

Each evaluation parameter was compared between the sexes using the Mann–Whitney *U* test. Pearson's correlation analysis was conducted to determine the correlation between BMI, FFMI, or SMI and other continuous variables. Multiple linear regression analysis was performed, with adjustment for age and sex, using BMI, FFMI, or SMI as response variables and seven oral‐related factors—oral moisture, tongue pressure, lip‐seal strength, oral diadochokinetic rate, and masticatory performance—as explanatory variables. We included 10 variables per subject in the multiple linear regression analysis because the present study used existing data. The number of teeth present was excluded from the explanation variables of the analyses because of the presence of multicollinearity between masticatory performance and the number of teeth present. EZR Version 1.37 (Saitama Medical Center of Jichi Medical University, Saitama, Japan) was used to perform all statistical analyses (Kanda, 2013). The significance level was set at *α* = 0.05.

## 3 RESULTS

### 3.1 Subjects

No patient was diagnosed with dysphagia via the repetitive saliva swallowing test (RSST). Table 1 shows the characteristics of the study subjects. BMI, FFMI, tongue pressure, and lip‐seal strength showed significant differences between sexes. Other parameters showed no significant sex‐based differences. The median number of teeth present was 27, with its distribution being non‐normal. The other elements showed normal distribution in their histograms. Among the 117 patients, 2 wore dentures and 1 had implant‐supported dental prostheses. The maximum tongue pressure (mean ± standard deviation [*SD*]) was 36.9 ± 7.9 kPa, and the lip‐seal strength (mean ± *SD*) was 13.6 ± 3.9 N in men and 12.0 ± 3.1 N in women. Of the 117 subjects, SMI was lower than the cutoff value in 23 subjects (19.6%).

**Table 1:** Characteristics of the subjects according to nutritional status and oral function

Measurement	Unit	Women (*n* = 73)			Men (*n* = 44)			*p*‐Value
		Mean ± *SD*	Median	Range	Mean ± *SD*	Median	Range	
Age	years	51.2 ± 6.5	52.0	40.0–64.0	49.2 ± 6.6	47.5	40.0–64.0	0.11
Nutritional status								
BMI	kg/m^2^	22.7 ± 4.2	22.0	16.5–34.6	23.7 ± 2.6	23.8	17.2–28.3	<0.01*
FFMI	kg/m^2^	15.6 ± 1.6	15.2	13.0–20.0	18.3 ± 1.5	18.3	14.7–20.7	<0.01*
SMI	kg/m^2^	8.4 ± 1.0	8.2	6.8–11.2	10.2 ± 1.0	10.3	8.0–11.7	<0.01*
Oral secretory function								
Mucosal wetness		25.3 ± 2.4	25.6	19.2–29.5	25.3 ± 2.4	25.9	18.6–29.5	0.97
Oral motor function								
Number of teeth present	n	25.3 ± 4.6	27.0	8.0–32.0	25.7 ± 4.2	27.0	8.0–29.0	0.72
Masticatory performance	mg/dL	176.9 ± 55.0	173.0	58.0–312.0	187.6 ± 64.4	199.0	38.0–340.0	0.19
Maximum tongue pressure	kPa	34.9 ± 7.0	35.0	13.4–49.8	40.1 ± 8.3	39.9	19.6–58.4	<0.01*
Lip‐seal strength	N	12.0 ± 3.1	11.0	6.8–21.2	13.6 ± 3.9	13.5	6.3–22.8	0.04*
Oral diadochokinetic rate								
/pa/	n/s	6.3 ± 0.8	6.4	4.0–7.8	6.5 ± 0.7	6.6	4.6–8.0	0.42
/ta/	n/s	6.7 ± 0.9	6.8	4.6–8.4	6.8 ± 0.8	6.8	5.4–8.2	0.99
/ka/	n/s	6.2 ± 0.7	6.2	3.2–7.8	6.1 ± 0.9	6.2	3.0–7.8	0.54

*Note*: Mann–Whitney *U* test; *p < 0.05.

Abbreviations: BMI, body mass index; FFMI, fat‐free mass index; *SD*, standard deviation; SMI, Skeletal muscle mass index.

**Figure 1:**
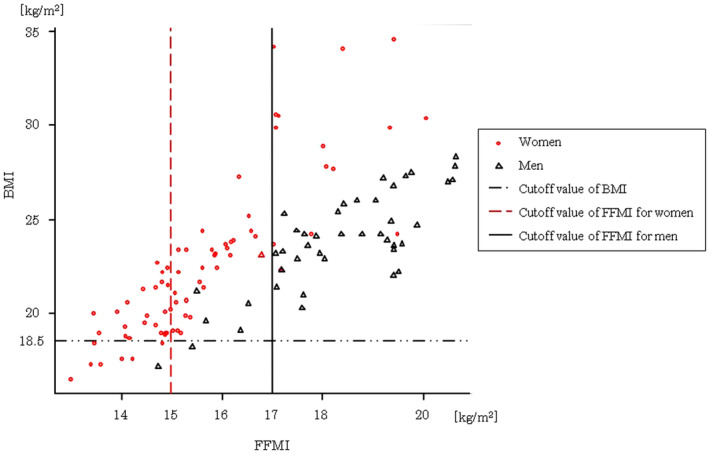
Scatter plot of the association between body mass index and fat‐free mass index. BMI, body mass index; FFMI, fat‐free mass index

The scatter plots showing the association between BMI and FFMI are presented in Figure 1. The cutoff value for BMI as per the ESPEN diagnostic criteria for undernutrition (Cederholm et al., 2015) was <18.5 kg/m^2^ and that for FFMI are <15 kg/m^2^ (women) and <17 kg/m^2^ (men). Of the 117 subjects, BMI was lower than the cutoff value in 9 subjects (7.7%) and FFMI was lower than the cutoff value in 36 subjects (30.8%).

### 3.2 Association between indices of body and muscle mass and factors related to oral function

Table 2 shows the results of Pearson's correlation coefficient analysis between nutritional status and factors related to oral function.

**Table 2:** Pearson's correlation coefficient between BMI, FFMI, or SMI and other continuous variables

Variable	BMI		FFMI		SMI	
	*r*	*p*‐Value	*r*	*p*‐Value	*r*	*p*‐Value
Age	−0.010	0.916	−0.106	0.254	−0.131	0.158
Oral secretory function						
Mucosal wetness	−0.052	0.578	−0.057	0.539	−0.053	0.574
Oral motor function						
Number of teeth present	−0.146	0.115	−0.072	0.444	−0.060	0.521
Masticatory performance	−0.048	0.611	0.032	0.731	0.034	0.717
Maximum tongue pressure	0.302	0.001*	0.417	<0.001*	0.423	<0.001*
Lip‐seal strength	0.261	0.004*	0.359	<0.001*	0.362	<0.001
Oral diadochokinetic rate						
/pa/	−0.010	0.916	0.134	0.151	0.139	0.136
/ta/	−0.139	0.136	−0.033	0.722	−0.020	0.831
/ka/	−0.103	0.268	−0.113	0.226	−0.111	0.235

Abbreviations: BMI, body mass index; FFMI, Fat‐free mass index; SMI, Skeletal muscle mass index.

Table 3 shows the results of the multiple linear regression analysis between nutritional status and factors related to oral function.

**Table 3:** Multiple linear regression analysis for the association with nutritional status

Variable	BMI (adjusted *R* ^2^: 0.097)					FFMI (adjusted *R* ^2^: 0.468)					SMI (adjusted *R* ^2^: 0.510)				
	B	*β*	*t*‐Value	VIF	*p*‐Value	B	*β*	*t*‐Value	VIF	*p*‐Value	B	*β*	*t*‐Value	VIF	*p*‐Value
Intercept	24.221		4.230		<0.001*	15.860		6.710		<0.001*	8.703		5.964		<0.001*
Age	0.022	0.039	0.431	1.066	0.667	0.001	0.002	0.031	1.066	0.975	−0.004	−0.019	−0.284	1.066	0.777
Sex [0: women,1: men]	0.406	0.054	0.550	1.185	0.578	2.210	0.543	7.363	1.185	<0.001*	1.507	0.576	8.134	1.185	<0.001*
Oral secretory function															
Mucosal wetness	−0.135	−0.087	−0.969	1.039	0.335	−0.075	−0.089	−1.293	1.039	0.199	−0.045	−0.084	−1.274	1.039	0.205
Oral motor function															
Masticatory performance	−0.009	−0.136	−1.422	1.180	0.158	−0.003	−0.076	−1.038	1.180	0.302	−0.002	−0.073	−1.032	1.180	0.304
Maximum tongue pressure	0.096	0.204	2.013	1.319	0.047*	0.039	0.156	2.000	1.319	0.048*	0.024	0.149	1.996	1.319	0.048*
Lip‐seal strength	0.265	0.252	2.467	1.341	0.015*	0.118	0.208	2.648	1.341	0.009*	0.073	0.200	2.656	1.341	0.009*
Oral diadochokinetic rate															
/pa/	−0.044	−0.009	−0.089	1.450	0.929	0.196	0.079	0.967	1.450	0.336	0.124	0.077	0.987	1.450	0.326
/ta/	−1.040	−0.234	−1.668	2.538	0.098	−0.183	−0.077	−0.710	2.538	0.479	−0.083	−0.054	−0.522	2.538	0.603
/ka/	0.480	0.104	0.730	2.598	0.467	−0.113	−0.045	−0.415	2.598	0.679	−0.095	−0.059	−0.565	2.598	0.573

*Note*: *p < 0.05.

Abbreviations: BMI, Body mass index; FFMI, Fat‐free mass index; SMI, Skeletal muscle mass index; VIF, variance inflation factor.

## 4 DISCUSSION

### 4.1 Key results

Multiple linear regression analysis of oral functions revealed that tongue pressure and lip‐seal strength, which are oral motor functions, were associated with nutritional status. Tongue pressure plays a vital role in bolus formation, and its reduction has been reported to be associated with decreased meal consumption (Namasivayam et al., 2016). Therefore, reduced tongue pressure may have reduced not only meal consumption but also BMI, FFMI, and SMI. Furthermore, both tongue pressure and lip‐seal strength are reportedly associated with sarcopenia (Sakai, Nakayama, Tohara, Kodama, et al., 2017). Poor nutritional status is a cause of sarcopenia, and insufficient energy intake results in decreased muscle mass (Cruz‐Jentoft et al., 2010). Therefore, a reduction in nutritional intake may induce sarcopenia, thereby resulting in reduced tongue pressure and lip‐seal strength.

### 4.2 Interpretations

The mean tongue pressure value calculated in the present study was similar to that reported in a previous study (Utanohara et al., 2008), in which the standard tongue pressure values in middle‐aged adults in their 40s and 50s were 40.4 ± 9.8 kPa and 40.7 ± 9.8 kPa, respectively. Subjects in the present study were patients who visited a dental clinic, and their mean number of teeth present was 25.4 ± 4.4. This indicates that the present study's population could be similar to healthy middle‐aged adults. Studies have also reported that factors related to oral function, such as the number of teeth present (Kikutani et al., 2013), occlusal force (Inomata et al., 2014), and masticatory function (Ikebe et al., 2006), are associated with the nutritional status of older adults. However, in this study, we did not find any association between masticatory performance and nutritional status. The median and mean ± *SD* of the number of teeth present in the subjects were 27 and 25.4 ± 4.4, respectively, which are greater than those in older adults, indicating that the occlusal force (Inomata et al., 2014) and masticatory performance (Morita et al., 2018) associated with the number of teeth present did not decrease and did not have any association with the nutritional status. Irrespective of whether the patients wore dentures or implants, masticatory performance assessed by eluate glucose concentration following gummy jelly chewing was used to evaluate masticatory function. Using this method, deterioration of masticatory function due to ill‐fitting dentures or tooth movement owing to periodontal disease can be comprehensively diagnosed. Hence, in the evaluation parameters in this study, we did not include information on whether the patient wore dentures or implants.

A recent study has reported on individuals with obesity and sarcopenia having high fat mass and low muscle mass (Cruz‐Jentoft et al., 2010). The rate of obesity increases in the middle‐aged and older populations compared with that in the younger population (Ministry of Health, Labour and Welfare, 2020). Lexell et al. (1988) reported that muscle mass loss becomes pronounced around the age of 50 years. FFMI‐based evaluation revealed more undernourished subjects than the BMI‐based evaluation. Therefore, we were able to extract the data of those subjects with high fat but low SM by evaluating both FFMI and BMI.

### 4.3 Limitations

The limitation of this study is that we did not investigate educational history and socioeconomic status (SES). Educational level of the subjects may affect nutritional intake. However, the impact of SES in this study may be small because health insurance covers dental treatment in Japan. In several previous studies, BMI alone has been used to evaluate nutritional status (Ikebe et al., 2006); however, with the addition of FFMI and SMI, we found that body composition analysis was useful for evaluating middle‐aged subjects. Also, FFMI and SMI were originally used in comparison to standard values, but we used them as continuous variables in this study, which is another limitation.

A history of cancer or deliberate weight loss may also affect BMI, FFMI, and SMI. Particularly, several middle‐aged adults intentionally lose weight. In the present study, none of the subjects had a history of cancer. Furthermore, we did not investigate the presence or absence of weight loss and intentional weight loss; hence, their effects on the results observed in this study remain unclear.

### 4.4 Generalizability

We found that tongue pressure and lip‐seal strength were associated with nutritional status of middle‐aged patients, even though the causal relationship was unclear because of the cross‐sectional study design and the fact that the subjects were from one dental clinic. Previous studies investigating the association between oral function and nutritional status included not only cross‐sectional studies but also quasi‐experimental studies and some cohorts, and a few factors associated with oral function were adopted by considering the association between oral function and nutritional status (Bertoli et al., 2014; Higashi et al., 2018; Ikebe et al., 2006; Inomata et al., 2014; Kikutani et al., 2013; Sakai, Nakayama, Tohara, Maeda, et al., 2017; Samnieng, 2015). Similar results were obtained in these studies, suggesting that these results can be generalized regardless of the study design. In the present study, multivariate analysis was performed with seven factors associated with oral function as explanatory variables. This analysis was performed with more than 10 subjects per explanatory variable. This approach may have enabled the evaluation of the association with nutritional status after adjusting for confounding factors among the several factors related to oral function. Previous studies have reported a tongue pressure of 40.4 ± 8.6 N in young adults (aged 21.9 ± 4.0 years; Tabuchi et al., 2018) and lip‐seal strength values of 14.2 ± 2.8 N and 12.6 ± 1.9 N in adult men and women, respectively (Saitoh et al., 2017), suggesting that tongue pressure and lip‐seal strength are retained with age. We identified linear correlations between nutritional status (i.e., BMI, FFMI, and SMI) and tongue pressure and lip‐seal strength, suggesting that nutritional status can be indirectly estimated by evaluating tongue pressure and lip‐seal strength.

## 5 CONCLUSION

Lip‐seal strength and tongue pressure were positively associated with BMI, FFMI, and SMI in middle‐aged patients of a regional outpatient dental clinic.

## CONFLICT OF INTEREST

The authors declare no conflict of interest.

## AUTHOR CONTRIBUTIONS

Tomotaka Nishi was involved in data collection and data analysis. Midori Ohta, Takayuki Ueda and Kaoru Sakurai were involved in study design and data interpretation. Tomofumi Takano and Koichiro Ogami were involved in data analysis and critical review. All authors critically revised the manuscript, commented on drafts of the manuscript, and approved the manuscript to be published, and agree to be accountable for all aspects of the work in ensuring that questions related to the accuracy or integrity of any part of the work are appropriately investigated and resolved.

## Data Availability

The data that support the findings of this study are available from the corresponding author upon reasonable request.
